# A Brief Review of Acoustic and Vibration Signal-Based Fault Detection for Belt Conveyor Idlers Using Machine Learning Models

**DOI:** 10.3390/s23041902

**Published:** 2023-02-08

**Authors:** Fahad Alharbi, Suhuai Luo, Hongyu Zhang, Kamran Shaukat, Guang Yang, Craig A. Wheeler, Zhiyong Chen

**Affiliations:** 1 School of Information and Physical Sciences, The University of Newcastle, Newcastle, NSW 2308, Australia; 2 Department of Information Technology, College of Computers and Information Technology, Taif University, P.O. Box 11099, Taif 21944, Saudi Arabia; 3 Department of Data Science, University of the Punjab, Lahore 54890, Pakistan; 4 School of Engineering, University of Newcastle, Callaghan, NSW 2308, Australia

**Keywords:** belt conveyor idlers, conveyor systems, deep learning, fault detection, machine learning

## Abstract

Due to increasing demands for ensuring the safety and reliability of a system, fault detection (FD) has received considerable attention in modern industries to monitor their machines. Bulk materials are transported worldwide using belt conveyors as an essential transport system. The majority of conveyor components are monitored continuously to ensure their reliability, but idlers remain a challenge to monitor due to the large number of idlers (rollers) distributed throughout the working environment. These idlers are prone to external noises or disturbances that cause a failure in the underlying system operations. The research community has begun using machine learning (ML) to detect idler’s defects to assist industries in responding to failures on time. Vibration and acoustic measurements are commonly employed to monitor the condition of idlers. However, there has been no comprehensive review of FD for belt conveyor idlers. This paper presents a recent review of acoustic and vibration signal-based fault detection for belt conveyor idlers using ML models. It also discusses major steps in the approaches, such as data collection, signal processing, feature extraction and selection, and ML model construction. Additionally, the paper provides an overview of the main components of belt conveyor systems, sources of defects in idlers, and a brief introduction to ML models. Finally, it highlights critical open challenges and provides future research directions.

## 1. Introduction

As industrial systems have become increasingly complex and expensive, performance degradation, productivity decrease, and safety hazards have become increasingly unacceptable. Belt conveyor systems are a critical component of many modern industries. They are perhaps the most extensively used transport method for conveying dry bulk materials in many production and manufacturing industries, such as coal, chemicals, electricity, steel production, and others [1,2,3]. Uninterrupted and trouble-free operation of belt conveyor systems is the compulsion of modern industries. As these systems have several components, a failure of one component can cause belt damage, economic loss, and death [4]. For example, a report from Brazil showed that from 2014 to 2016, fires caused by conveyor idler failures resulted in losses of approximately AUD one million, in addition to 600 h of downtime [5]. Potential faults and abnormalities in such systems must be identified and detected as soon as possible to minimize performance degradation and prevent dangerous situations before a sequence of damage can be catastrophic. Numerous factors can result in the causes and damage of a belt conveyor, including the working conditions, continuous movement, contact with rotating components, and material transport [6]. These causes and damages in belt conveyor systems have led to the development of intelligent data-driven fault diagnosis and detection systems with the aid of machine learning (ML) models that use vibration [4], thermal [7], and acoustic [8] methods to detect faults in belt conveyor systems.

A fault diagnosis is defined as determining the type, size, and location of the fault, the time of fault detection, and the behavior of the fault in accordance with the appropriate assessment of the faults [9]. Fault detection is an essential component of fault diagnosis, which involves identifying or indicating faults in a process or system and then isolating the faulty process or variable to obtain additional useful information concerning the faults [10,11]. The effect of the early detection of faults in belt conveyor systems can lead to a minimum amount of downtime and maximized production. However, the traditional fault detection of belt conveyor systems is manual, requiring workers to inspect rotating machinery such as rollers [12] regularly. In addition, some conveyors are very long, several kilometers long, and it can be challenging to manually inspect the entire conveyor length [13]. As a result, it is time-consuming and labor-intensive to perform manual FD, which will not allow the fault to be found in time due to the reliance on subjective measurements by maintenance operators. The methods of FD have evolved rapidly in recent years from manual detection to intelligent detection using ML models.

In intelligent FD, machine learning theories are applied to detect machine faults [11]. This can be characterized as a classification problem or a pattern recognition problem in order to release human labor and automatically recognize a machine’s state of health, which is why it has attracted significant attention in the last two or three decades. Although intelligent FD has achieved a substantial number of successes, a review of the development of intelligent FD in belt conveyor idlers leaves a blank space and rarely provides guidelines for future development. For example, Qurthobi et al. [14] provide a systematic review of acoustic approaches to mechanical failure detection, including 52 studies of recent implementations and structures focusing on all rotating machinery without emphasizing specific components such as idlers. Wei et al. [15] present a review and summary of research on early fault diagnosis of gears, rotors, and bearings. In their review, however, idlers were not mentioned. An additional review [11] was conducted that focused on the task of diagnosing failures in rotating machinery in general. As a result, the evidence accumulating from previous research is not well documented regarding belt conveyor idlers. Furthermore, a review is necessary due to the unique characteristics of belt conveyor idlers and their spatial distribution. These include:

- Idlers are subject to high levels of wear and tear due to the constant contact with the conveyor belt and the heavy loads they support. This can lead to the development of unique faults such as misalignment, shell wear, damage to the rollers, or structural deformation [16].
- Idlers typically have low rotational speeds, making it more difficult to detect faults using traditional monitoring methods that are typically used for higher-speed machinery [17,18].
- Idlers are typically exposed to harsh environments, such as dust, heat, and moisture, which can accelerate the degradation of the components and increase the likelihood of faults [19].
- Idlers have a simple construction and are low-cost components, which means that there is a high number of them in a conveyor system. This can present challenges for monitoring and diagnosis, as many idlers need to be inspected regularly.
- Idlers have an important role in the conveyor system, supporting the belt. Therefore, the early detection of faults in idlers is crucial to prevent belt damage and to ensure the conveyor system runs reliably [16].
- Idlers are part of a complex system, and they are affected by the dynamic behavior of the conveyor system, which can vary depending on the specific application, operating environment, conveyor design, and loads [20].

These peculiarities of idlers make a review specifically focused on fault detection in idler belt conveyors important, as it can help to identify the most common types of faults, the most effective methods for detecting them, and any challenges or limitations associated with monitoring these systems, which are specific to idler belt conveyors.

**Figure 1:**
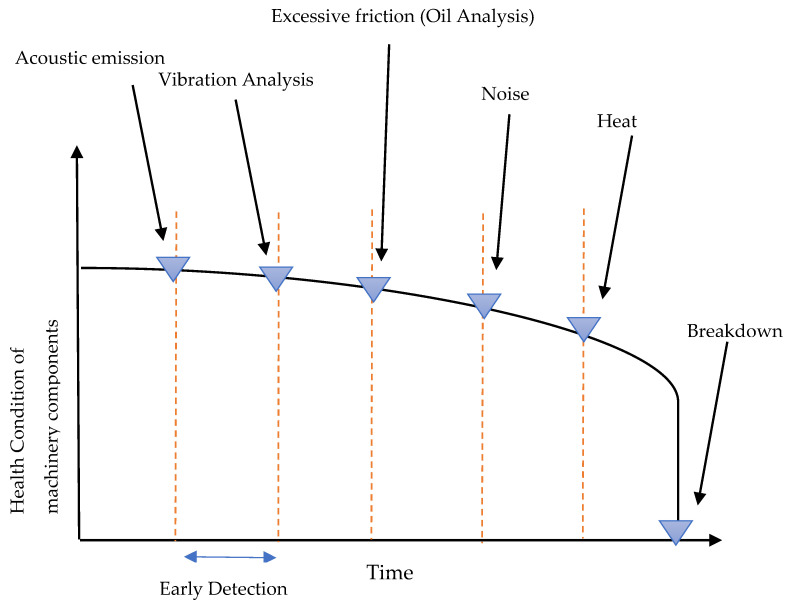
The level of detection for each method of monitoring.

Researchers have focused on developing FD systems for rotating machinery using vibration and acoustic signals over the last two decades [4,21]. This is because vibrations and acoustic signals provide better indicators of conveyor belt damage in the early stages. Figure 1 shows the level of detection for acoustic and vibration methods for detection machinery components such as idlers [22]. Based on the figure, it can be seen that vibration and acoustic information can provide superior signatures for detecting early faults to other methods, such as heat or thermal imaging methods. Further, there are a number of articles published recently on vibration and acoustics methods that demonstrate the effectiveness of both methods in the detection and monitoring of belt conveyor idlers. Acoustic and vibration methods are widely believed to be the most effective in detecting faulty idlers [4,8]. Observing the vibration responses of belt conveyor idlers can reveal their state, and vibration analysis is widely used for fault detection [23]. A vibration sensor must be used to capture the state of idlers, which is a contact sensor that can be difficult to install [24]. Compared to vibration, acoustic recording is a safe and non-destructive method for capturing the sound of faulty idlers. While they are both affected by the position of the sensor, both can detect multiple idlers simultaneously and detect a wide range of defective idlers. As a result, researchers prefer both for detecting faulty idlers.

Using heat or thermal imaging to detect failing idlers is another approach. As the roller rotates, friction occurs between its internal parts. An idler’s bearing is an essential component that, if malfunctioning, will increase friction and temperature [1]. By utilizing a hand-held thermal imaging camera, faulty idlers can be detected by an operator. However, since the belt conveyor is typically located in a harsh environment, there is a lot of dust on the site, which can interfere with the image information and potentially distort the reading [24]. On this basis, thermal image-based detection methods are not considered accurate and reliable methods for intelligent FD, and will not be considered in this review. It is also important to note that thermal image-based detection methods are limited in terms of the number of articles published [25,26,27]. Table 1 compares vibration-based methods with acoustic-based and thermal image-based methods in terms of five characteristics: early detection, detection of a wide range of faults, accuracy, detection of multiple idlers simultaneously, and effects of environmental conditions.

### 1.1. Contribution of the Paper

To the best of our knowledge, there has been no comprehensive review of acoustic and vibration signal-based FD for belt conveyor idlers using ML models. Therefore, this paper is the first to provide an overview of the research on FD tailored to belt conveyor systems. This review aims to assist other researchers, particularly those new to FD of belt conveyor idlers, to identify gaps in the literature, evaluate reports critically, and establish the foundation for developing ML-based detection models for diagnosing idlers based on acoustic and vibration signals. In addition, we hope it facilitates a better understanding of the different directions in which research has been performed on this topic, and how current ML state-of-the-art models developed. The main contribution of this paper is summarized as follows:

- An overview of the components of belt conveyor systems, sources of defects, and a brief introduction to ML models.
- A segmentation of the whole program of FD systems into four blocks, i.e., data acquisition, signal processing, feature extraction and selection, and ML models for fault detection.
- A review of ML models used in FD for belt conveyor idlers based on acoustic and vibration signal-based methods.
- Discussion on several challenges in detecting failures in belt conveyor idlers and highlights of future research directions.

### 1.2. Information Sources and Search Strategy

This review covered the years up to October 2022 and was conducted using the electronic database Scopus and Web of Science. It is well known that Scopus provides a more extensive collection of peer-reviewed articles [28]. A search of Google Scholar was undertaken to cross-check and forward search the findings and citations and to locate other papers in less well-known libraries. Our selection of these databases was based on their ability to provide full-text access to the most important peer-reviewed journals and conference proceedings in machine learning.

**Figure 2:**
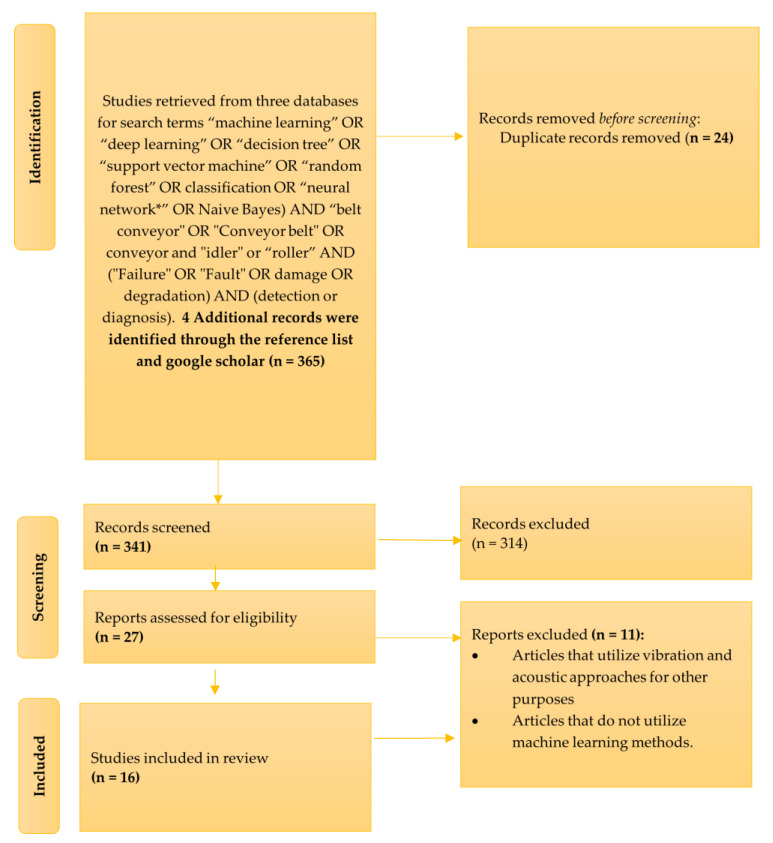
An illustrative view of the process for article selection.

A bibliographic search was conducted using search terms for three concepts in the article’s title, abstract, and keywords: (1) ML technology was represented as (“machine learning” OR “deep learning” OR “decision tree” OR “support vector machine” OR “random forest” OR classification OR “neural network*” OR Naive Bayes), (2) belt conveyor idlers was coded as (“belt conveyor” OR “Conveyor belt” OR conveyor?) AND (“idler” or “roller”), (3) failure detection was coded as (“Failure” OR “Fault” OR damage OR degradation) AND (detect* OR diagnose* OR “condition based maintenance” OR “predictive maintenance” OR prognostics). These search terms were combined into a search query using conjunction AND disjunction OR operators. Figure 2 illustrates the selection process for articles.

The search strategy described above resulted in a total of 365 articles being retrieved. A total of 27 of these papers were selected for full-text assessment, and 16 of these papers met the inclusion criteria, as shown in Table 2.

### 1.3. Organization of the Paper

**Figure 3:**
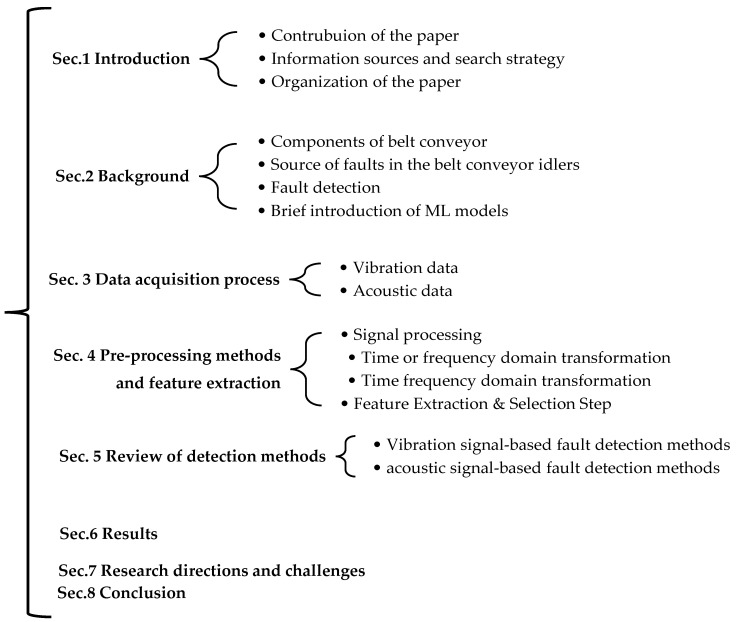
Outline of this paper.

The overall organization of this paper can be seen in Figure 3. This paper is organized as follows: In Section 2.1, the belt conveyor components are discussed. Section 2.2 demonstrates the fault sources and the type of faults in belt conveyor idlers. Section 2.3 presents a brief review of the stages of FD. Section 2.4 provides an overview of ML models used to detect faulty idlers. The data acquisition process for idlers on belt conveyors is presented in Section 3. Section 4 determines what pre-processing methods and features are useful for developing FD systems for detecting faulty idlers. Section 5 reviews the detection methods used in the context of FD systems in belt conveyor idlers using vibration and acoustic signals. Section 6 describes a set of new research directions and challenges concerning the idlers. Finally, in Section 7, we present our conclusion.

## 2. Background

This section provides a background on the key components of belt conveyor systems, the source of faults and a brief introduction to ML models. A list of acronyms used in this article is provided in Table 3.

### 2.1. Components of a Belt Conveyor

**Figure 4:**
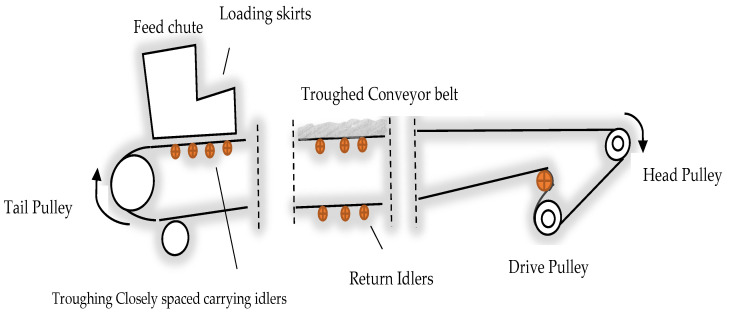
Structure of a belt Conveyor.

The belt on roller conveyor systems is considered one of the many conveyor systems available [29]. These systems consist of several continuously monitored components to maintain their reliability. Figure 4 illustrates the belt on roller conveyor systems with a belt spanned between a head and a tail pulley [30]. The head pulley is connected to a drive unit that consists of an electric motor, multiple couplings, and a gearbox. One of the most important components of a drive unit is the gearbox [31]. According to [32], 14% of gearboxes need to be replaced yearly due to unexpected failures. Wear or damage to the geared wheels (broken teeth or excessive backlash) and bearing failures (due to fatigue of outer/inner races and rolling elements) are the causes of these failures.

**Figure 5:**
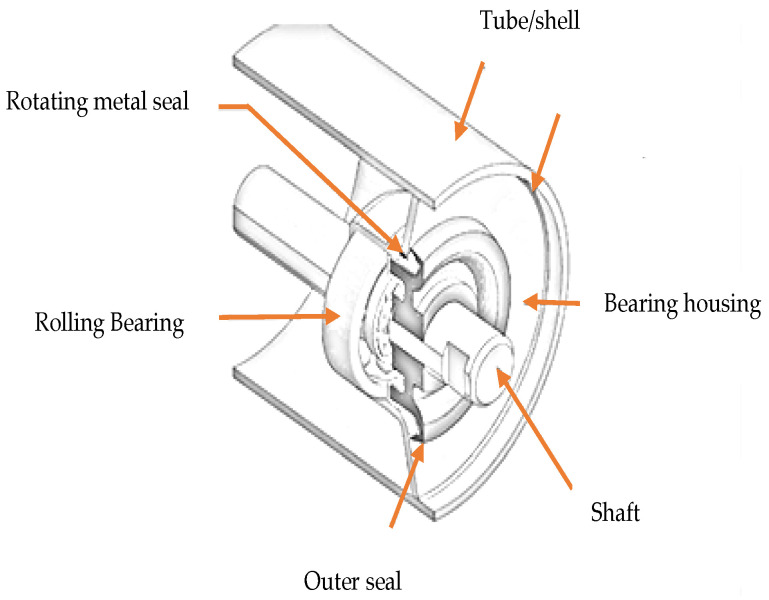
Structure of belt conveyor idler.

In order to support the load of the bulk material on the belt, spatially distributed carrying idlers support the belt along its length [17]. A report indicates that more than half of conveyor belt failures are caused by malfunctioning idlers [33]. The idler is one of the most common rotating components in belt conveyor systems. Load-bearing idlers are also referred to as return and carry idlers [2]. Each idler roller consists of a shell, shaft, two bearings and housings, and two sealing systems. Figure 5 shows the typical structure of belt conveyor idlers. A significant part of the roller’s performance is the bearings, considered one of the most important components of the roller that contribute to the reliable operation of the idler rolls [34]. To better understand the belt conveyor idlers components, it is necessary to understand the type of faults that arise from such components.

### 2.2. Source of Faults in the Belt Conveyor Idlers

**Figure 6:**
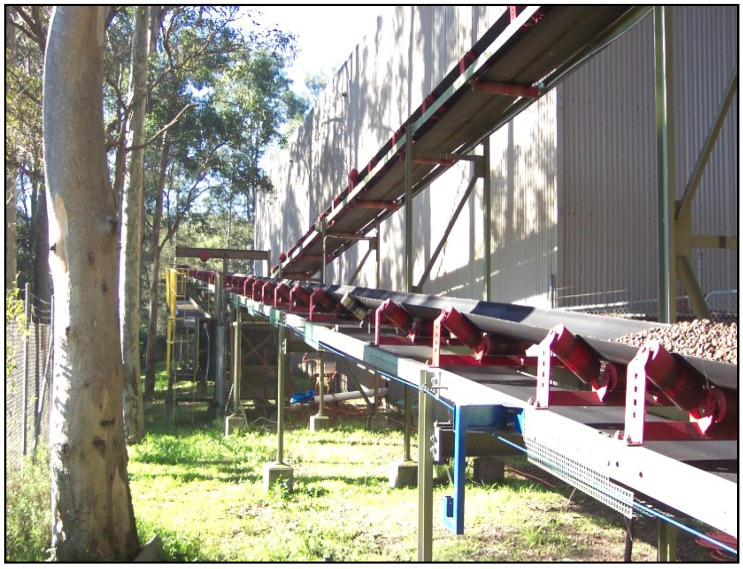
Idler rolls are shown in operation on a typical belt conveyor.

Due to their rotating nature, when functional failures occur in components of belt conveyors, such as idlers, pulleys, and gearboxes, they generate noise that can be measured vibrationally or acoustically [19,35]. Idlers are generally believed to be the primary noise source in belt conveyor systems in acoustic and vibration methods [19,33]. Figure 6 illustrates the use of idler rolls on a typical belt conveyor.

**Figure 7:**
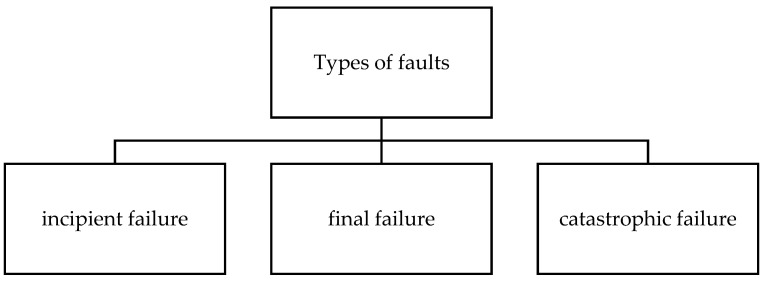
Types of failures in belt conveyor idlers.

Idler roll failures can be classified into three types: incipient failure, final failure, and catastrophic failure, as depicted in Figure 7 [36]. A large amount of the literature on the FD of belt conveyor idlers falls between the stages of incipient failure and final failure. According to [36], incipient failure refers to spalling or fatigue on the bearings that occurs when rolling elements wear out due to pitting, fretting, abrasive or adhesive wear. Before the incipient failure occurs, bearings in rollers are functioning correctly and are in a healthy condition. The rollers will last as long as the bearings last [37]. Then, bearing fatigue occurs when rolling elements wear out due to pitting, fretting, abrasive or adhesive wear. Factors such as lubrication, dust, and moisture can affect the bearings [38,39]. These incipient failures can lead to or develop into final faults, such as impermissible noise emission, excessive runout, a seized roller, or even bearing collapse states.

For example, Liu et al. [8] investigated the incipient failures acoustically of three types of artificially defected bearings, including the damaged bearing, cage, and cover, which have been found to cause serious and final conveyor system failures. Using the audio-based method, Peng et al. [24] studied the roller in the context of three types of failures. Similarly, rollers have been studied [40,41] in binary classification between normal and faulty conditions based on several scenarios, including broken rollers, rotation off-center, collisions, and friction between the belt and the roller. Different approaches have been taken to study the rollers using robots [42,43] which successfully detected the defective idlers. Roos and Heyns [23] used a vibration signal method to investigate incipient faulty idler bearings at three lateral positions on the belt: near the faulty bearing, in the middle of the belt, and over the healthy bearing. Another study [44] analyzed a vibration signal anomaly caused by a defect in the roller bearing, friction between the belt and the blocked tracking roller, and abrasion of the roller tube. Several other studies by Ravikumar et al. [45,46,47,48] investigated the vibrations of an incipient faulty idler as part of the multiclassification task to train different ML models. Idlers that are incipiently failing can still perform their functions until they fail. Several rollers must be observed to prevent belt failures from causing a fire and causing a catastrophic event. Catastrophic failure refers to idlers that have failed severely and cannot function properly, which may result in severe damage to belt conveyor systems, such as fire. Idlers in the final or catastrophic failure stage will produce more thermal infrared radiation due to the increase in resistance [7]. Idlers can generally be affected by three major defects: breaking, overheating, and seizing.

### 2.3. Fault Detection

A fault detection system (FD) for industrial processes is designed to produce an effective indicator that can identify faulty processes and prevent future failures or unfavorable events [9]. For example, the petrochemical industry loses approximately 20 billion dollars annually due to faults in its machine components [49]. Likewise, according to a report, approximately 60% of the cost of aircraft engine components is attributed to maintenance [49]. In the worst-case scenario, a malfunctioning machinery component may result in the death of a human being.

The process of diagnosing faults can be divided into four stages: fault detection, fault identification, fault severity assessment, fault growth and remaining useful life prediction [22]. The process of fault detection involves the examination of the components of machinery for faults. Based on the previous section, most research on fault detection is concerned with incipient faults in idlers to enable the next stage of the FD process to be performed. A fault identification process involves locating and identifying the type of fault in machinery components. Wijaya et al. [12] proposed a combination of a statistical analysis based on vibrational signal and unsupervised ML models to determine the fault location of belt conveyor idlers. The size of the fault determines the severity of a fault in the belt conveyor idler. This can be estimated by extracting statistical information from idlers. In the remaining useful life (RUL) process, the life cycle of a machine component is predicted. A fault’s growth is predicted based on the size of the fault after a certain number of cycles. RUL analysis predicts the breakdown period/time of the component. In an FD system, this analysis might be beneficial if the output RUL prediction is expressed as a function of time and fault size. Our investigation indicates that most of the literature has been devoted to studying the first stage of fault diagnosis. Therefore, the review will focus on fault detection since studies on fault identification, fault severity, and useful life prediction have not yet been completed regarding belt conveyor idlers.

### 2.4. Brief Introduction of ML Models

FD systems in belt conveyor idlers have been widely designed based on various supervised and unsupervised machine learning algorithms: Random forests (RF) [50], K nearest neighbors algorithm (*K*NN) [40], Support vector machines (SVM) [40], Gradient boost decision tree (GBDT) [8], and Isolation forest (IF) [12]. Models of this type are sometimes referred to as shallow machine learning models. The FD systems based on shallow machine learning models are called traditional FD systems based on statistical data-driven. It consists of four primary steps, as indicated in Figure 7: acquisition of data, processing of data, extraction of features, selection and reduction of dimensions, as well as the classification of features. Identifying appropriate methods for each step requires a trial-and-error approach. The massive number of features in the model must be carefully selected to reduce the computational complexity, thereby preventing degradation of the classification accuracy when used in traditional FD systems.

**Figure 8:**
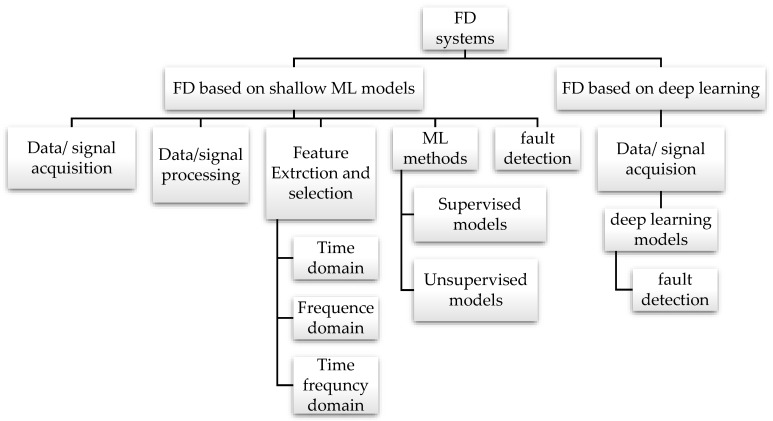
The difference between deep learning and shallow machine learning models in FD systems.

The supervised learning method is a subset of ML models that tries to find the target label from a set of predictive variables. It uses the training dataset to achieve the desired results [51]. After identifying patterns, the algorithm creates a model to generalize the acquired rules to new data sets. Random forests (RF), K-nearest neighbors algorithm (*K*NN), support vector machines (SVM), and decision tree examples of these methods have been applied in the literature. A significant weakness of supervised methods is that sufficient label data may not always be available to train models in FD [52]. This is because rotating machinery, such as idlers, typically function in normal conditions, making it difficult to have sufficient labeled data that includes faults. In addition, conditions make it difficult to collect samples of faults. Thus, in the absence of fault data, it is difficult to collect samples as idlers can consider different conditions, noisy samples, and environmental changes. Unsupervised models are, therefore, an ideal alternative. *K*NN is an example of unsupervised ML models used in FD in belt conveyor idlers. It uses feature similarity to predict the values of new data points [11]. The value of each point will be determined by how closely it matches the training points. The isolation forest algorithm is another unsupervised ML model used to detect anomalies. Instead of modeling the normal points, this method detects anomalies based on isolation (the distance between a data point and the rest of the data). Figure 8 shows the difference between shallow machine learning and deep learning algorithms in FD systems.

On the other hand, acquiring labeling data is a huge cost [11]. This led to the other types of ML models, which are unsupervised ML models. Unlike the supervised model, unsupervised ML models provide unlabeled data that the models have by their tries, which does not rely on such expert knowledge in feature extraction and pattern recognition [51]. The idlers of belt conveyors generally operate under healthy conditions.

Models based on deep learning do not require a combination of methods for each step in the FD process; the process is carried out by utilizing multiple hidden layers in the model’s architecture. It emerged as a highly effective network structure for feature extraction and pattern recognition in fault detection [11]. Due to its more powerful and sophisticated feature extraction capabilities, deep learning has shown better results than shallow approaches in the fault detection of belt conveyor idlers. ML models that have been proposed in the literature for detecting idlers are Multilayer perceptron (MLP) [50], Convolutional neural networks (CNN) [24], and Autoencoder (AE) [40]. These methods may also be applied to other rotating machinery, such as gearboxes [53], pumps [54], and turbines [55], etc., to detect and classify faults. A comparison of the strengths and limitations of each machine and the deep learning model used in the FD of belt conveyor idlers is presented in Table 4.

## 3. Data Acquisition of Belt Conveyor Idlers

**Figure 9:**
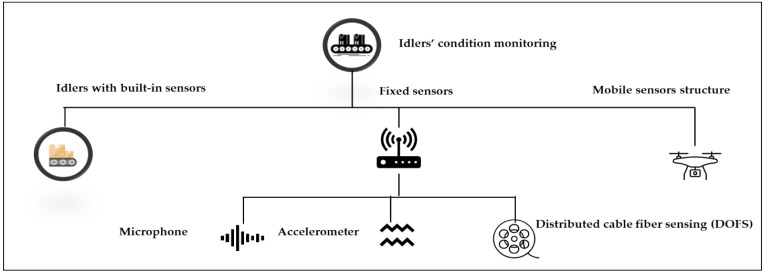
Data collection methods.

The data acquisition process for idlers on belt conveyors is presented in this section. Two types of sensor data are discussed here, including acoustic data and vibration data. In assessing idlers’ conditions in FD systems, the type and position of sensors play a critical role. The types of sensors can be categorized as follows: built-in sensors, fixed sensors, and mobile sensor structures, as shown in Figure 9. The input data in FD systems are shown in Table 5 according to the type of idlers sensor used.

### 3.1. Acoustic Data

It has been proposed in the literature that several methods can be used to collect acoustic data from idlers. For example, Ericeira et al. [50] collected data using an ultrasonic device and trained RF and MLP algorithms using the time and frequency domain statistical features. Another study [41] used statistical features from the generated sound signal to train a neural network to detect defective idlers. According to Table 6, the dataset and the method for collecting data using acoustic signals are described.

Since microphones are relatively inexpensive and flexible to install in industries, acoustic data can easily be collected [56]. For example, Liu et al. [8] used a microphone to collect sound and investigate a combination of statistical features with Mel Frequency Cepstrum Coefficients (MFCC) to train Gradient Boost Decision Tree (GBDT) as a classifier for fault detection on belt conveyor idlers. MFCC features in audio analysis, such as speech recognition, have proven to be highly reliable [57]. Another study used a microphone and statistical features from the frequency domain to train the RF model. In both studies, the type of microphone used was not mentioned, and the amount of data collected was limited to training ML models in FD systems.

**Table 5:** Input data in FDD systems.

Input Types	Vibration Methods	Acoustic Methods
Raw input domain	[44]	
Statistical features from the time domain or frequency domain	[45,46,47,48,58]	[8,50,59]
Statistical features from the time-frequency domain	[4,12,23,60]	[24,41]
Combination of statistical features from all domains		[40]

**Table 6:** Datasets and methods for data collection using acoustic signal.

Authors	Dataset	Method for Data Collection
Rocha et al. [59]	The final dataset contains 55 audio files	Microphone
Ericeira et al. [50]	Ten time-series in non-defective idlers and ten recordings for the defective ones. Each recording lasted for approximately 20 s	ultrasonic sensor
Liu et al. [8]	42 sets of acoustic data acquired from experiments, which is equal to 420 s of sound data acquisition.	microphone
Yang et al. [40]	Not reported	Sensor
Peng et al. [24]	A total of 8000 sets of sample data are collected	Sensor
Xiao-ping Jiang and Guan qiang Cao [41]	Not reported	Generated sound signal

The complexity of the acoustic signal collected by a fixed sensor highly depends on how the sensor is attached to idlers. It is less flexible to attach and requires a greater maintenance level than microphones. Yang et al. [40] collected the data from six sensors and combined the statistical features from multiple domains to train various ML and deep learning algorithms. Peng et al. [24] also used sensors to collect data, which was then analyzed statistically using time-frequency domain features to train CNN.

Mobile sensor structures have also been used recently, which utilize a sensor dedicated to belt conveyor idlers, enabling continuous detection of faulty rollers. These two studies [42,43] developed a robot that moved within a conveyor belt frame to measure the acoustic signal from the rollers and then analyze the data through advanced signal processing techniques. However, despite promising results, neither study has implemented ML models that would help detect faulty idlers.

### 3.2. Vibration Data

Due to legibility and intuitiveness, vibration data has been the most common source of information in FD systems. Most studies for FD systems used fixed sensors to collect vibration data. For example, Muralidharana et al. [58] used an accelerometer sensor to collect data for training a decision tree based on statistical features. Roos and Heyns [23] used time-frequency features to identify and classify faulty idler bearings based on SVM and ANN. Ravikumar et al. [46] used statistical features from data collected by accelerometer sensors to train artificial intelligence and Naïve Bayes. The same authors train several ML models using statistical features from vibration signals, including SVM, RF, and K star algorithms [45,47,48]. These studies just collected 1250 samples, and the experiments were performed and tested in the laboratory.

A vibration sensor must be used to capture the state of idlers, which is a contact sensor that can be difficult to install [24]. Therefore, Li et al. [4] applied one acceleration sensor to collect vibration signals from several idlers to reduce the number of sensors. A fault detection system was then trained using statistical features. An additional study by Bortnowski et al. [44] used a wireless measuring device, which moves along the conveyor belt along the route, to record the signal of transverse vibrations of the belt. Another study proposed a built-in sensor enclosed in the idlers to capture vibration signals as an Internet of Things (IoT) device [29]. Integrating a sensor within the roller allows remote monitoring of individual rollers. This kind of sensor can be integrated with ML models to detect faulty idlers in real-time.

A specific number of accelerometers will not be sufficient to monitor an entire conveyor, and the cost of such a system is probably too high to justify. Therefore, it is possible to monitor the condition of mining conveyors in real-time with distributed optical fiber sensors (DOFS). The DOFS can measure temperature, strain, and acoustic signals by using a relatively thin optical fiber in the cable as the sensor. These sensors are sensitive to external perturbations, thus the name distributed sensor. For example, Wijaya et al. [12] developed an anomaly detection method using DOFS signals and statistical features. Another study by the same author [60] captured the vibration signals using DOFS technology and then processed them using ANN based on frequency analysis of the faulty idlers. As a result of the DOFS technology, there is no need to use multiple sensors for long and distributed infrastructures since the optical fiber cable itself acts as a continuous sensor. Table 7 describes the dataset and the methods used to collect data using vibration signals.

## 4. Signal Processing and Feature Extraction

This section explains two key steps in the FD processes in the first stage: signal processing and manual feature extraction and selection. Researchers usually combine all of these stages when implementing deep learning algorithms.

### 4.1. Signal Processing

The processing of a signal, whether acoustic or vibration, refers to transforming the signal into a representation that can be used to develop a ML model. Due to the long vibrational or acoustic signal length, a very long signal cannot be processed in one instant in FD systems. Therefore, windowing in FD systems is commonly employed to segment the signals into several segments [22]. For analysis, the segmented signal is then divided into training and testing samples to train ML models. Several signal processing and analysis-based methods are successfully used in belt conveyor idlers, which aim to obtain the original features by the signal transformation, such as the wavelet transform (WT) [24,41,61], fast Fourier transforms (FFT) [8,12,50,59], local mean decomposition (LMD) [42], and envelope analysis [12], etc.

Wavelet transformation is a time-frequency domain feature that uses wavelets as its basis rather than sinusoidal functions [62]. It adds a scale variable to the time variable in the inner product transform. Since the wavelet transform can zoom and have adaptive windowing capabilities due to its variable time localization and frequency resolution, it is particularly suited to nonstationary signal analysis [15]. The energy distribution of the respective bands and the energy changes over time were analyzed in [24] using the wavelet packet transform on the signal to decompose detailed information of the high-frequency and low-frequency components of the signal. Other examples [41,61] used wavelet transforms to detect fault sound characteristics represented by a low-frequency band. The difference between wavelet transforms, and wavelet packet decomposition (WPD) is that the wavelet transform applies the wavelet transform step to the low pass result, while wavelet packet transform applies the wavelet transform to both the low pass and high pass result of the signal.

The FFT algorithm converts the time domain signal into a frequency spectrum with frequency resolution [59]. For example, Liu et al. [1] used Fourier transform to analyze the experimental data collected from idlers and obtained the sound frequency band (FB), which can effectively detect the final failures of idlers. In some cases, it may be necessary to average these frequency bands during the data recording period to remove any non-periodic signals, such as impacts of tools or materials, that could interfere with the frequency data [12]. There is also the option of local mode decomposition (LMD), employed as a denoising method [42]. The process involves adaptively decomposing the signal into product functions (PFs), which combine an envelope signal with a frequency-modulated signal. Then a spectral autocorrelation map (SAC) is calculated for each denoised signal produced by LMD. In this way, the cyclic behavior of a signal can be observed in the carrier frequency spectrum, and the frequency of idler rotation can be determined, which indicates the failure of a bearing in idlers.

Envelope analysis is one of the techniques used to process the signal, which is widely used to extract fault information from rotating machinery, particularly bearings. A fault produces an impulse in a time-domain signal. In response to the impulse, the structure’s natural frequencies are excited, which are determined by the structure’s dynamic properties. Traditional frequency analysis can identify this natural frequency component, but it is impossible to identify the repeated impulse. Therefore, it is more advantageous to use envelope analysis to determine the impulse repetition frequency than direct FFT, as direct FFT is generally controlled by the resonance frequency, which is often concealed by the high-order smearing [12].

The processing may include other types of pre-processing, such as normalizing, filtering, and removing a certain signal length of signals [8,40,44]. The normalization process transforms the signal amplitude to be in the range of −1 to 1, which is conducive to FD systems. For example, in this study [41], after obtaining the features extracted from WT, they normalized the energy for each layer. They took them as the signal’s feature vectors to train the neural network. Filtering the noise is another way to pre-process the signal in FD systems. For example, in this article [44], the authors used a three-stage input signal filtering: wavelet decomposition, one-dimensional median filter, and Butterworth low-pass filter algorithms were employed to reduce the interference.

### 4.2. Feature Extraction & Selection Step

A feature in FD systems is a parameter derived from the measured data capable of robustly indicating the presence of belt conveyor idler faults [57]. In other words, it is the process of focusing on the most discriminating signal characteristics, representing a useful tool in FD systems. The next step is to select a subset of sensitive features related to belt conveyor idlers’ health states based on a number of factors, such as correlation, principal component analysis (PCA), stacked autoencoder [40], and Pearson correlation [60]. The performance of the ML models is determined by these steps [63]. Features, in general, can be sub-categorized into three main categories: time domain, frequency domain, and time-frequency domain [10].

#### 4.2.1. Time or Frequency Domain Transformation

Statistical information in the time or frequency domain is used as input information for ML models [22]. Time domain signal processing is generally used for FD of components where fault severity produces periodic shocks/peaks in the time domain signal [31]. This transformation uses scalar indices and is based on the value of the temporal vibrational signal data to estimate the idler’s condition. For example, statistics such as the root mean square (RMS), skewness, kurtosis, and PAK, which is the ratio of the peak value of sample data to its RMS value, are used to analyze the time-domain features of sound signals [7]. They are prominently distinguished from that of other faulty idlers, while that of the remaining faulty idlers are almost indistinguishable from that of the intact one.

Using the time domain, we can observe how the signal changes over time [57]. The time domain features can be categorized into dimensional and dimensionless features [11]. The former include mean, standard deviation, root amplitude, root mean square, peak value, and variance, which are affected by the speed and load of belt conveyor systems. The latter mostly includes shape factor, skewness, kurtosis, crest indicator, clearance indicator, and impulse factor, which are robust to the operation conditions of the belt conveyor systems. For example, Liu et al. [36] verified by experiment using the dimensional feature, RMS values of vibration signals to detect final failures of idlers. Other time-domain features include zero crossing rate (ZCR) and autocorrelation-based features, the former being defined as the rate of change in the sign of a signal used to estimate its fundamental frequency.

Autocorrelation measures the self-similarity between a signal and its delayed version in the time domain [43]. Researchers used to study the signal further by utilizing the autocorrelation feature to detect periodic peaks [43]. The signal could be correlated with another signal due to cross-correlation analysis. The work in [22] examined cross-correlation analysis of the original signals to detect the fault idler to determine whether peak information can be acquired in the delay domain. However, it is difficult to determine the accuracy of fault detection and classification algorithms by using only one feature. Therefore, in this study [60], several commonly used statistical time domain features (e.g., RMS, peak to peak, standard deviation, skewness, crest factor, and kurtosis) have been considered. Other articles [45,46,48,58] used statistical features as a part of the analysis, including mean, median, mode, standard error, standard deviation, kurtosis, skewness, minimum value, maximum value, sample variance, and range with different ML models.

Based on [58], it can be concluded that the time domain features of idler sounds are not useful for distinguishing incipient, final, and catastrophic corrosion faults but should only be used to detect corrosion faults with obvious abnormal sounds. Therefore, time domain features are considered unreliable indicators of early FD of idlers compared to frequency because they contain limited fault information [7,50]. Table 8 provides a brief description of the most common features in the time domain as well as their formulas.

Similarly, frequency domain or spectral analysis is widely used to detect and diagnose idler faults. FFT is employed here to convert vibration signals in the time domain into discrete frequency components to detect changes or faults in the belt conveyor idlers [50]. It is one of the most crucial signal analyses because it contains information that cannot be obtained from time-domain features. For example, Wijaya et al. [12] performed an FFT analysis every second, providing a frequency resolution of one Hz. The frequency data is then averaged over the data recording period. The envelope analysis is then used to determine the impulse repetition frequency since the resonance frequency typically governs direct FFT, often obscured by smearing harmonics of higher order. Table 9 provides an overview of vibration signal pre-processing and feature extraction.

Another feature is Mel frequency cepstral coefficients (MFCC), which compactly represent the frequency spectrum when it represents a waveform by summating an infinite number of sinusoids of an audio signal. It involves two domain changes: from the time domain to the frequency domain (FFT) and back to the time domain. MFCCs have equal frequency bands, which are very similar to the human auditory system, making them a key feature in various audio signal processing applications. Regarding fault detection, MFCCs have been implemented on belt conveyor idlers and have produced excellent results [8,40]. According to [7], we conclude that the frequency domain characteristics of idler sound signals are suitable for detecting final and catastrophic defects resulting from corrosion, radial overload, or cage or rolling element damage. However, these features cannot detect faults caused by pitting, fretting, or abrasive wear. Since the frequency domain is limited, this leads to a time-frequency domain that combines both time and frequency. An overview of the preprocessing and feature extraction of acoustic signals is presented in Table 10.

#### 4.2.2. Time-Frequency Domain Transformation

When a signal is analyzed using a time-frequency transform, the signal is viewed from the perspective of time on one axis and frequency on the other [57]. Consequently, time-frequency analysis is an effective tool for detecting signals’ frequency components and revealing their time-varying features, which have proven useful in monitoring and fault detection. This processing method aims to convert the belt conveyor idlers’ time series into 2D time-frequency representations. Short-time Fourier transform (STFT) is one example of time-frequency features that computes several Fourier transforms at various intervals in order to preserve information about time and the evolution of sound over time [8]. This study [7] examined STFT using Fourier transforms. As a result of STFT, a 2D matrix is created, which is a time-translation variable, transforming the signal into spectrograms. These spectrograms are visual representations of a signal’s spectrum of frequencies as they change over time, which is implemented in [43]. It consists of a two-dimensional matrix with an *x*-axis representing time, a *y*-axis representing frequency, and a value representing signal intensity, usually represented as a colored chart.

Wavelet analysis is another time-frequency feature that effectively translates one-dimensional vibration signals into two-dimensional time-frequency domains. It allows recognition of non-stationary features within the signal as well as extracting time-frequency-domain signals, which are not possible for frequency domain analysis, such as FFT and envelope analysis. An important factor in wavelet analysis is the type and order of the mother wavelet functions. Daubechies wavelet (Db) is widely used as a mother wavelet function in analyzing vibration signals from rotating machinery [64,65]. A Db wavelet is denoted by the notation DbN, which refers to the number of vanishing moments. Considering theoretical analysis and practical experience, the authors in [4] have selected Daubechies (db5) wavelet basis function as it is considered more suitable for fault signal analysis for rotating machinery than other basis functions.

Another study [60] used four decomposition levels to obtain 24 features, analyzed according to their correlation with the output, and filtered by Pearson correlation to eliminate features with low correlations. Db9 is selected as the optimal wavelet function in the analysis, which illustrates that the key issue is selecting the appropriate number of vanishing moments. It is important to note that wavelet analysis is essentially a Fourier transformation with an adjustable window. The decomposition of a signal with wavelet is limited to the rectangular time–frequency partitioning of the time–frequency plane, and such tile partition does not guarantee that the instantaneous frequencies of the resulting components obtained by wavelet transformation have physical significance. Therefore, wavelet analysis lacks a feature of self-adaptation. Overall, Liu et al. [7] verified in experiments that time-frequency domain features of idler sound signals can distinguish final and catastrophic problems caused by corrosion, cage, and rolling element damage, radial overloading, and idler blocking, as well as larger size bearing problems caused by pitting, fretting, abrasive or adhesive wear. Still, it is more difficult to distinguish small-size bearing problems in idlers.

ML models rely on feature selection as part of the processing of the signal, and the techniques used can have substantial effects by focusing on relevant attributes and reducing noise. For example, Ravikumar et al. [46,48] used a decision tree to select features. Based on the experimental results, they found that kurtosis, standard deviation, and mean values of vibration signals are effective features for fault detection of belt conveyor idlers. A second study [60] used Pearson correlation to select features for training ML models. The authors claimed that this method identifies individual variables’ contributions to the output variance. A different study [40] reduced the dimensions of suspected fault data using the PCA algorithm. Based on the examples listed, this process significantly impacts the accuracy of the final ML models.

## 5. Review of FD Methods Based on Shallow Machine Learning and Deep Learning

Many variants of supervised and unsupervised ML models have been studied and applied to the FD of belt conveyor idlers. Thus, this section will review these publications according to the methods used. In Table 11, the source of each paper selected is listed, along with more information about each one. According to the table, idlers have been a significant research topic in the past ten years. The most extensive research was focused on vibration methods for detecting fault idlers, with ten articles utilizing ML models based on different extracted features. Acoustic methods, however, have not progressed at the same rate as vibration methods. Acoustic methods for detecting faulty idlers are currently limited to six articles.

### 5.1. Vibration Signal-Based Fault Detection Methods

ML models can be fed segmented signals in vibration or acoustic form. When detecting faults in idlers, measuring the vibration signal and performing FD methods is intuitive and effective. A list of vibration FD methods for belt conveyor idlers can be found in Table 12. According to the table, almost all of the methods that have been proposed have achieved an accuracy of more than 90%. In most studies, traditional machine learning models are developed, with a few employing deep learning algorithms. In most studies, relatively small data sets were used, and incipient faults, which are the most common causes of idler bearing failure, were examined.

Many researchers in the FD field highly prefer SVM as it produces significant accuracy with less computation power [66,67]. In this article [40], an SVM algorithm was developed for detecting idlers by detecting different types of faults, and the detection accuracy was 91.9%. Another example has been proposed by Li et al. [4] to implement an online monitoring and FD system for idlers based on WPD and SVM, where vibration signals of idlers are collected using acceleration sensors. As a result of the study, it was found that the wavelet coefficients of the vibration signals were effective features for detecting idler faults. Another study by Ravikumar et al. [47] developed SVM to discriminate between normal and non-normal Self-aligning carrying idler (SAI) conditions to detect SAI faults automatically. Results of the experiment indicate that the method was effective for detecting faulty idlers with an accuracy rate of 98.08%. An investigation of the dynamics of a payload conveyor was conducted using SVM and an artificial neural network (ANN) to detect bearing faults using WPD [23]. SVM was classified at 100%, while ANN was classified at 98.00%, demonstrating the superiority of SVM. The SVM model is effective for handling small numbers of monitoring data. The massive data, however, is difficult to fit, which may result in the curse of computation. Secondly, SVM-based detection models are sensitive to the parameters of the kernel. As a result of the inappropriate kernel parameters, reliable detection results cannot be obtained.

In order to improve the accuracy of classification greedily, the GBDT model combines Gradient Boosting and Decision Trees to produce a single model. Gradient boost achieves accurate classification rules efficiently by minimizing its loss function greedily using decision tree ensembles as base learners. It is believed that the GBDT model, with sufficient training data, can detect faulty idlers with an accuracy of 94.53% [8]. Muralidharana et al. [58] used statistical features and a decision tree algorithm for detecting idler faults. Several experimental results demonstrate that statistical features, including kurtosis, standard deviation, and mean values of vibration signals, are effective for detecting idler faults. GBDT provides great flexibility since it can be optimized on various loss functions, and the hyperparameters can be tuned to give the function as much flexibility as possible. It also accurately predicts faults by considering a variety of hyperparameters. However, GBDT is computationally expensive because they require many trees to train the detection model, which takes a significant amount of memory and time.

The random forest (RF) method is another Ml method proposed in the literature. Compared to a decision tree, a random forest reduces the risk of overfitting using multiple decision trees instead of one [68]. For example, Rocha et al. [59] developed RF to effectively identify damaged bearings noise in belt conveyor idlers with an accuracy of 95%. Another study [50] used RF with a trial-and-error approach in extracting features and tuning the hyperparameter of the number of trees. The results obtained by this study were satisfactory in detecting the abnormalities in idlers. RF was also used in [48] to categorize digital vibration signals after extracting effective statistical features. It was able to categorize faults correctly with an accuracy of 90.2%. The RF algorithm produces good predictions that are easy to understand; however, if there are a large number of trees, the algorithm becomes too slow and inefficient to make real-time predictions. It is important to note that supervised ML models rely heavily on labeled inputs and outputs, which are usually difficult and expensive to collect in real industries. It will facilitate unsupervised ML models, which are based on unlabeled data.

In addition to previous methods, *K*NN is also examined to detect faulty idlers using vibration signals. It uses feature similarity to predict the values of new data points [11]. Each point will be assigned a value based on how closely it matches the points in the training set. In this study [40], different K values and clusters were set to different numbers. It has been found that the best experimental results of more than 90% accuracy are obtained when the k parameter is set to 10. It is relatively simple to implement the *K*NN-based detection models. However, to handle the large volume dataset, *K*NN requires much computation. Another disadvantage of this model type is that it would have a lower detection accuracy because the data distribution would be imbalanced, which is the case when detecting idlers [11]. Additionally, it is difficult to determine the parameter k, which has a significant impact on the performance of the model.

The isolation forest (IForest) algorithm is used to detect anomalies or abnormal points from the dataset. For instance, a modified isolation Forest method has been proposed in [12], which is considered an unsupervised algorithm for detecting most anomalous data points with a 90% improvement in analysis time. Instead of modeling the normal points, this method detects anomalies based on isolation (the distance between a data point and the rest of the data). As a result, IForest does not rely on distance or density measurements to identify anomalies, resulting in greater than 90% detection accuracy compared to manual inspection on-site. Another technique is principal components analysis (PCA), which has been used to reduce dimensionality and is considered one of the famous unsupervised techniques that take correlated variables stored in the dataset and generate a set of new variables with no linear correlation based on compressing that dataset [40]. However, unsupervised ML models sometimes result in undesirable results due to a lack of label datasets.

A CNN model is a type of deep learning proposed for detecting idlers. This supervised deep learning model has been examined in [24]. In order to input features into CNN, the authors used mean and standard deviation as features. The classification accuracy rate of the mean is 86%, and the classification accuracy rate of the standard deviation is 93%. This shows that using standard deviation as a data feature is more effective in detecting idler faults. As a result of CNN’s capability to capture shift variant properties, recognition models based on CNN can be used without any processing, such as frequency-domain transformation. Moreover, by sharing weights in detecting models, the number of training parameters can be reduced, accelerating convergence and preventing overfitting. There is a need to train CNN-based detection models with sufficient labeled samples to enhance their detection performance.

Autoencoder (AE) is also proposed in the literature. Bortnowski et al. [44] developed an LSTM autoencoder, one variation of autoencoders, to automate the detection of anomalies in recorded diagnostic signals using designated time series. Initially, the research was conducted in a laboratory setting using a roller with prepared damage. Afterward, the adopted test procedure was validated under real-world conditions. Limitations of the method include identifying specific types of damage faults and assessing the detection method in real-life situations. The representational power of AEs is limited due to their simple and shallow structure. It is possible to stack multiple AEs in a configuration called stacked AEs, which can increase the representational power by utilizing the values of the hidden units of one AE to influence the values of the subsequent AEs used in this study [40]. Based on the logarithmic loss function minimization and automatic feature extraction using the key characteristics of stacked AEs, the average accuracy was 94.4%. The experiment results indicate that the deep neural network is more accurate than the shallow algorithm regarding recognition. Even though AE is an excellent tool for capturing the characteristics of highly complex and nonlinear patterns, it has also been shown to learn to capture the characteristics of information not specifically required for detecting those patterns.

Vibration measurement via DOFS is a potential method of real-time monitoring of mining conveyors at a reasonable cost. It is known as a distributed sensor since the fiber in the cable acts as a sensor itself and is sensitive to external perturbations. As a result of the technology, temperature, strain, and acoustic signals can be measured. For example, Wajia et al. [60] employed the WT to evaluate and analyze the characteristics of vibration signals obtained from the DOFS. For the detection and classification of damage, an ANN model is implemented for the development of a smart fault detection technology. Under varying conveyor operating speeds, the proposed method has 99% accuracy in classifying damage conditions. However, the experimental setup has the limitation of not being able to eliminate the effects of background noise on conveyors and simulate the loading effects on conveyor frequency signatures. In another study by Ravikumar et al. [46], an idler FD algorithm using ANNs and Naive Bayes algorithms was proposed. Experimental results demonstrate that NB performs better than ANN when trained and verified using idler vibration data.

### 5.2. Acoustic Signal-Based Fault Detection Methods

Acoustic signals have been used to detect faults in belt conveyors in various ways in the literature. Several mining studies have been conducted to inspect belt conveyor structures [42] and to detect a longitudinal tear in conveyor belts [69,70], which have achieved good results in acoustic signal-based fault detection. In recent years, more research has been focused on acoustic-based FD methods for idlers. In both the time and frequency domains, statistical features have been used to detect faults in idlers. For example, Liu et al. [7] proposed a method to analyze the faults in belt conveyor idlers using the features of thermal infrared images (TII) and sound. A statistical method was used to analyze the time-domain features of sound signals and the thermal infrared image features, while a quantitative method based on Fisher’s linear discriminant was used to analyze the frequency-domain and time–frequency-domain features. According to the authors, incipient bearing faults and final catastrophic faults can occasionally be detected using the frequency-domain features of idler sound. Another study [71] investigated a frequency domain feature called Teager energy spectral kurtosis. This feature integrates the Teager energy operator with the impulse characteristic and the sensitivity of the frequency domain statistical index to periodic components. The proposed method was verified to be more efficient in identifying bearing fault sounds and locating the source of idler-bearing fault sounds.

Another way to detect abnormal idler sounds is through ML models. Ericeira et al. [50] presented a methodology for detecting early failures in belt conveyor idlers. Several experiments were conducted with time domain and frequency domain data and different attribute vectors to determine which preprocessing and feature extraction methods were more effective for enhancing classification performance. Based on the results of various classification experiments using RF and MLP, the ultrasound spectrum displays a distinctive pattern that distinguishes non-defective idlers from defective idlers. Coa and Jiang [41] focused on roller fault sound audio analysis, exploring a new kind of automatic FD method based on WT and backpropagation neural network technology, as well as a method of removing noise to extract fault features to improve system recognition accuracy. Current FD methods using acoustic signals suffer from the following limitations: the types of idler faults are insufficient, the selection of acoustic features is arbitrary, and most neglect the interference of running noise from the belt conveyor. For example, A FD method based on sound signals was developed in this study [8]. In order to classify the data, they used MFCCs as features and the GBDT algorithm. Even though the test accuracy and recall rate are satisfactory, sound data are limited, and the proposed approach needs to be examined in the context of data with intense background noise. Peng et al. [24] proposed an audio-based intelligent fault detection method for idlers. The wavelet coefficients of idler sound are used as the input of CNN to classify idler roller sticks and fracture faults. The result of the experiment indicates that the detection method is accurate, fast, and robust, which significantly improves the efficiency of fault detection of sand carrier rollers. However, the number of samples the model trained on is considered limited. Audio-based intelligent fault detection methods for belt conveyors were studied using a different machine and deep learning algorithms in this paper [40], showing some good results in inspecting roller running states in the coal industry. According to the running results, the fault detection system is extremely effective for detecting roller faults, with an accuracy rate of 96.7%. A list of acoustic FD methods for belt conveyor idlers can be found in Table 13.

Since there are a large number of idlers to monitor, the conveyor is large, and there is a risk of accident when dealing with rotating elements and moving belts, monitoring all idlers is not practical from both a scale and connectivity perspective [34]. Shiri et al. [42] proposed the use of an inspection robot to capture acoustic signals for condition monitoring. The condition of the idler was then assessed through the use of signal processing techniques, local mode decomposition (LMD), and a spectral autocorrelation map (SAC) was calculated for each denoised signal produced by LMD that helps pre-process and analyze the signal. The mobile inspection robot could automatically detect cyclic modulations in the audio recording to determine whether the idler had a mechanical problem. Rocha et al. [59] developed a ground robot that consists of a mobile platform, robotic arm, and sensor set for inspecting conveyor structures over long distances. By processing visual, thermal, and sound data as inspection functions, ML models detect dirt build-ups on conveyors, roller failures, and bearing faults with detection accuracy superior to 90%. Using a single robot will not be sufficient for long conveyors. A possible solution is using ROSI robots and drones to cover large conveyor sections quickly and rail-guided devices to operate in difficult access areas.

Skoczylas et al. [43] presented a robotic solution to detect the incorrect operation of roller sets, which support the conveyor belt that transports the material. The authors proposed an acoustic diagnostic feature to detect the incorrect operation of belt conveyor idlers based on the spectrum of the acoustic signal. The experiments were conducted in industrial conditions, which were not the expected environment for most of the algorithms used in the comparison. In another paper [12], the DOFS system was proposed to effectively detect faults on mining conveyors. Statistical features of high-frequency energy were employed as the primary parameter for determining the fault in the developed detection framework. The proposed framework has been evaluated in a field trial and has demonstrated greater than 90% detection accuracy compared to manual on-site inspections. Recent studies indicate that other methods for collecting acoustic signals need to be developed.

The development of other acoustic methods has also been widely used in fault detection for various industrial systems, such as motors [72], pumps [73], drills [74], and gearboxes [75]. SMOFS and MSAF are both frequency methods in which fault features are automatically selected based on the differences in frequency between the spectrum components [76]. MSAF (Method of Selection of Amplitudes of Frequencies) is a hand-crafted method that is specially designed to detect faults in electric motors used for drilling or grinding [57,77]. The method extracts the differences between FFT spectra of good and faulty signals and computes the absolute values of these differences to find common frequency components. These frequency components are then grouped and used to form a feature vector for classification purposes. SMOFS (Shortened Method of Frequency Selection) is similar to MSAF in that it is used to analyze audio signals and determine which frequencies are crucial for identifying various states of a signal based on a threshold [57]. Both methods require the selected parameters, and the number of groups is determined before testing. MSAF-17-MULTIEXPANDED-FILTER-14 [78], SMOFS-22-MULTIEXPANDED [79], MSAF-RATIO-24-MULTIEXPANDED-FILTER-8 [80], and MSAF-RATIO-27-MULTIEXPANDED-4-GROUPS [72] are some variations of the MSAF and SMOFS methods, which have different parameters and algorithms, depending on the type of the fault and the target system. For example, MSAF-17-MULTIEXPANDED-FILTER-14 specifies that the method looks for one to 17 common frequency components, and 14 refers to the 14 Hz bandwidth used to determine a feature vector [78]. An additional example of a different parameter is SMOFS-22-MULTIEXPANDED, in which 22 refers to the number of frequency components used in feature extraction [81]. These frequencies are selected from a range of acoustic signals and then analyzed to detect faults in electric motors. The advantage of these methods is that they are simple, efficient, and effective in detecting various types of faults. However, the disadvantages of these methods are that they may be sensitive to noise, vibration, and other factors that affect the quality of the signals, and they may require some adjustments and optimization to achieve the best performance. Listed in Table 14 are the advantages and disadvantages of each method.

## 6. Results

The results of acoustic signals were compared with those of vibration signals in the literature. The result shows that acoustic detection methods have the advantage of detecting subsurface cracks, whereas vibration detection methods detect defects only when they are visible on the surface [82]. In another study [83], acoustic signals were shown to be highly effective for the early detection of faults in gearboxes. They may be an effective tool for identifying the various types of faults progressing. It is important to note that the vibrational energy released by neighboring components within the vibrational frequency range (up to 50 kHz), which often masks the vibrational energy released by defective rolling element bearings, does not have any effect on the acoustic signal released at very high frequencies [82]. Additionally, acoustic signals are airborne, making them a very advantageous communication medium. Therefore, a microphone is sufficient to capture the signals, whereas, in vibration monitoring, a sensor must be mounted directly or indirectly on the machine under observation; therefore, errors may be caused by the misalignment of the sensor [84].

Based on the review, some of the results of this study include the following:

- ML models, such as artificial neural networks and support vector machines, can improve fault detection accuracy for belt conveyor idlers compared to traditional methods.
- ML models can analyze large amounts of data from multiple sensors, including vibration, acoustic, and thermal data, to detect faults in belt conveyor idlers.
- ML models can identify patterns in the data that indicate a fault, such as changes in vibration amplitude or frequency, which can help diagnose faults in the early stages.
- ML models can also be used to classify the different types of faults, such as bearing wear or misalignment, which can help to optimize the maintenance and repair of the conveyor system.

The Table 15 below summarizes the reviewed ML techniques, their capability to detect different types of idler faults, and their importance in belt conveyor systems. A fault is considered important if it results in a faulty device; otherwise, it is viewed as less important. The effectiveness of ML models was evaluated based on their ability to detect different types of faults, handle real-world data, and be applied to various studies. An assessment of the efficiency of the ML models is described using levelsone to five, where level five refers to the model applied to more than one study, considered a real-world industry, and faults investigated are deemed important.

## 7. Challenges and Future Research Directions

FD has achieved a large number of successful applications on vibration signal-based methods. However, due to the mixture of background noise and sound, less attention has been paid to acoustic for FD systems of belt conveyor idlers. Data access is also an issue in building acoustic-based signal ML models in FD systems. The availability of standard datasets with associated tasks has improved the capabilities of learning systems in multiple tasks in different ML tasks, such as image recognition [85], speech recognition [86], natural language processing (NLP) [87], etc. However, progress appears to lag in some fields, such as FD in belt conveyor idlers. This is due to belt conveyor systems’ complex, noisy environment and the lack of acoustic and vibration datasets. Furthermore, rotating machines, such as idlers, generally work in healthy conditions [9], making FD difficult. Therefore, further research regarding the role of FD using acoustic and vibration signals would greatly help find practical solutions and improve the literature. The following are some topics that should be studied by researchers in depth for FD applications for belt conveyor idlers:

1. Research on multi-information fusion and multi-model [88] approaches should be investigated. This is because multivariate versions can extract a greater amount of fault information, and in real-world applications, multiple channels of signals are measured simultaneously. Therefore, weak fault symptoms can be detected at an early fault stage; utilizing balanced health states can reduce missed diagnosis rates and improve detection accuracy [67].
2. There is a lack of research addressing how the results can be generalized to dynamic settings and studies on how belt conveyors can be used under changing conditions. As part of the current data-driven approach to FD, the training and testing data must be in the same working condition and have the same distribution and feature space. As a result, future studies are recommended to improve test results’ reliability since most of the literature has been conducted in laboratory settings, and they may not be able to incorporate all types of data in real-world applications.
3. Research on the development of interpretable models. As a result of the use of ML models, there is often a concern that these methods are black boxes, and it is unclear how certain decisions are derived. Thus, in order to understand the type of faults that occur in certain machines, interpretable models must be constructed.
4. Using Unmanned Aerial Vehicles (UAVs) to inspect critical industrial components, such as idlers on belt conveyors, has proven to be a very interesting method of solving accessibility and mobility issues. The drone’s rotors and engines produce echo noise, which can cause a problem. In order to extract hidden information from a signal, complex signal analysis methods are required. A wide range of studies have been conducted on UAV noise [89,90]
5. Research on the lack of labeled sound data. With the availability of large datasets, Deep Learning solutions can provide significant benefits in the modeling process. It should be noted that a major milestone in computer vision has been achieved with the availability of a massive labeled dataset, resulting in the birth of ImageNet [85].
6. Research on model evaluation metrics. In most studies, accuracy (ACC) was used as a metric for evaluation. However, it is important to note that ACC is not the appropriate measure for imbalanced classification problems that commonly occur in belt conveyor idlers. The model can achieve a very high ACC if it can predict the majority class correctly (non-failure), even when it performs extremely poorly in predicting the minority class (failure). It is, therefore, necessary to differentiate between the accuracy of minority and majority classes, for which precision, recall, and specificity, among other metrics, are available for evaluating ML models.

The detection of faults is an important area of future research. Suitable action may be taken to prevent further degradation and serious damage to the products if the faults arising in the processes can be detected or predicted properly. Furthermore, in the era of big data, it is imperative to develop FD strategies that are real-time and comprehensive, utilizing all the information at hand.

## 8. Conclusions

This review provides a comprehensive overview of the published research utilizing ML models to detect belt conveyor idlers’ failures. It focuses on vibration and acoustic methods since they detect early defects effectively. Presented in the review are the components of belt conveyor idlers, the sources of faults, the processing techniques, the feature extraction methods, and a variety of ML models and deep learning used in FD for belt conveyor idlers. The paper has paid attention to acoustic and vibration signal-based fault detection using ML models for belt conveyor idlers. Several ML models, such as ANN, *K*NN, SVM, decision trees, and deep learning, have been discussed in detail. The use of deep learning algorithms such as CNN has a significant impact on the accuracy of the detection process. It shows promising results when it comes to detecting idler faults. Results of the review indicate that barriers to developing ML-based failure detection models and their performance evaluation continue to exist. This is due to a lack of public datasets for idlers, which makes it difficult to accumulate knowledge about ML-based failure detection models and to select the appropriate parameters for FD. In addition, since idlers are distributed all over the belt, we suggest that drones are used to detect defects in belt conveyor idlers automatically. Overall, we believe this review will enhance knowledge about the use of machine learning for failure detection for belt conveyor idlers.

## Figures and Tables

**Table 1:** Comparison of different methods used for idlers detection (legend: √ means a method has this feature, and x means it does not).

Method	Early Detection	Detect a Wide Range of Faults	Accuracy	Detect Multiple Idlers Simultaneously	Affected by Environmental Conditions
Vibration-based methods	√	√	x	√	√
Acoustic-based methods	√	√	x	√	x
Thermal image-based methods	x	x	x	√	√

**Table 2:** Inclusion and exclusion criteria.

Inclusion Criteria
Articles proposing a machine learning method mainly or hybrid with other methods for belt conveyor idler failure diagnosis and detection
2. Articles that utilize or validate machine learning methods for FDD in belt conveyor idlers
3. Articles demonstrating FD for belt conveyor idlers written in the English language only
**Exclusion Criteria**
Articles that are not written in English
2. Articles that utilize acoustic and vibration approaches for other purposes
3. Articles that do not utilize machine learning methods
4. Articles providing unclear results or findings
5. Duplicated studies

**Table 3:** List of acronyms.

**AE**	Autoencoder
**ANN**	Artificial neural network
**CNN**	Convolutional Neural Network
**DOFS**	Distributed optical fiber sensors
**FD**	Fault detection
**FFT**	Fast Fourier transforms
**GBDT**	Gradient boost decision tree
**IForest**	Isolation Forest
***K*** **NN**	K-nearest neighbors algorithm
**LMD**	Local mean decomposition
**MFCC**	Mel Frequency Cepstrum Coefficients
**ML**	Machine learning
**MLP**	Multilayer perceptron
**PCA**	Principal component analysis
**RF**	Random Forest
**RMS**	Root mean square
**STFT**	Short-time Fourier transform
**SVM**	Support Vector Machine
**WPD**	Wavelet packet decomposition
**WT**	Wavelet transform

**Table 4:** A summary of ML models in idlers detection using vibration and acoustic signals.

Models	Strengths	Limitations
**Random Forest (RF)**	RF algorithm is capable of handling large data sets with a high degree of dimensionality. RF produces good predictions that are easy to understand.	RF is primarily limited by the fact that the algorithm becomes too slow and inefficient for real-time predictions if there are a large number of trees.
**K-nearest neighbors algorithm (*K*NN)**	During the process of making real-time predictions, it does not have a training period and only makes use of the training dataset while making predictions in the future. *K*NN is easy to implement since only two parameters are required, namely K and the distance function.	It does not perform well when dealing with large datasets. It is not suitable for large dimensions.
**Support Vector Machine (SVM)**	It is more effective to use SVM in high-dimensional spaces. There is a relatively low memory requirement for SVM.	It is not recommended to use SVM algorithms for large datasets. This is due to the high training complexity of SVMs.
**Gradient boost decision tree (GBDT)**	There is a great deal of flexibility—it can be optimized on various loss functions and has several options for tuning hyperparameters to make the function fit as flexibly as possible. It is able to predict faults with a high degree of accuracy.	The computational cost of GBMs is high as they often require many trees (more than 1000), which takes a great deal of time and memory.
**Convolutional Neural Network (CNN)**	It requires little pre-processing, reducing the human effort required to develop its functionalities. A good performance was achieved when extracting local features from images.	There is a need for a large amount of training data. Training is computationally intensive.
**Isolation Forest (IForest)**	A small sample size is more effective. A low memory requirement and minimal computational effort. Scalable to handle extremely large data sets and multidimensional problems with a large number of irrelevant attributes.	Has difficulty finding anomalous items that are closely surrounded by ordinary items. Does not perform well when an ordinary item is in close proximity to an anomalous item.
**Autoencoder (AE)**	A great tool for extracting features. Suitable for representing highly complex and nonlinear patterns.	Rather than capturing as much relevant information as possible, learns to capture as much information as possible.
**Multilayer perceptron (MLP)**	It is applicable to complex nonlinear problems. Ability to handle large amounts of data. Predict after training on time.	Model performance depends on the quality of the training data.

**Table 7:** Datasets and methods for Data Collection using vibration signal.

Authors	Dataset	Methods for Data Collection
Bortnowski et al. [44]	Not reported.	Wireless measuring device.
Li et al. [4]	32 samples under each health condition are obtained, each load case with 8 samples. Hence, there are a total of 128 samples.	Accelerometer sensor.
Wijaya et al. [12]	The data size can reach up to 60 MB for 1 s signal.	DOFS.
Muralidharan et al. [58]	Collect 250 samples for each condition of the Self-aligning carrying idler.	Accelerometer sensor.
Wijaya et al. [60]	3000 samples were collected.	DOFS.
Roos and Heyns [23]	Dataset of 3007 training and 3007 test signals.	Accelerometer sensor.
Ravikumar at al. [45,46,47,48]	Collect 250 samples for each condition of the Self-aligning carrying idler.	Accelerometer sensor.

**Table 8:** Most common time domain features and their formula.

Time Domain Feature	Definition	Formula	Remark
Root mean square (RMS)	It is used to compute the average energy of the signal.	$RMS=\sqrt{\left(\frac{\sum_{n=1}^{N}X^{2}\left[n\right]}{N}\right)}$	N = sample size. n = single value of N. X = amplitude value of sample size. μ = mean value of sample data. σ = standard deviation. Xpeak = maximum peak value of X. X_RMS_ = root mean square value.
Standard deviation	Represents the degree of variation or dispersion from the average.	$\sigma ={\left(\frac{1}{N}\sum_{n=1}^{N}{\left(X-\mu \right)}^{2}\right)}^{1/2}$	
Kurtosis	It is dimensionless and the fourth-order normalized moment of sample data sensitive to impact signal and represents the steepness of the data distribution density function.	K = $\sqrt{(\frac{\frac{1}{N}\sum_{n=1}^{N}{\left(X\left[n\right]-\mu \right)}^{4}}{{\left(X\left[n\right]-\mu \right)}^{2}{)}^{2}}})$	
Skewness	This is a dimensionless indicator of the degree of asymmetry of the data distribution as represented by the third-order normalized moment of the data distribution.	S = $\;\frac{\frac{1}{N}\sum_{n=1}^{N}{\left(X\left[n\right]-\mu \right)}^{3}}{\frac{1}{N}\sum_{n=1}^{N}{\left(X\left[n\right]-\mu \right)}^{3}{)}^{\frac{3}{2}}}$	
Peak to the average value (PAR)	It is defined as the ratio of the peak to the average value of a sample of data and can be used to indicate significant transient noise.	PAR = $\frac{X_{Peak}}{X_{RMS}}$	

**Table 9:** Pre-processing and feature extraction for vibration approach.

Authors	Processing Techniques	Feature Extraction
Bortnowski et al. [44]	Filtering and normalization using the mean.	Spectrogram, signal autocorrelation (ACF), mean peak frequency.
Li et al. [4]	WPD.	The energy of each frequency band is extracted as the feature
Wijaya et al. [12]	Fast Fourier transform.	Envelope analysis.
Ravikumar et al. [45,46,47,48]	Trimmed off to ensure the uniform length of the signal.	The various parameters are mean, median, mode, standard error, standard deviation, kurtosis, skewness, minimum value, maximum value, sample variance, range.
Wijaya et al. [60]	Wavelet transform.	Extracted features from WT and statistical time-domain features (e.g., RMS, peak-to-peak, and standard deviation)
Muralidharan et al. [58]	Trimmed off to ensure the uniform length of the signal.	The various parameters are mean, median, mode, standard error, standard deviation, kurtosis, skewness, minimum value, maximum value, sample variance, range.
Roos and Heyns [23]	WPD.	A sum of squares of the frequency magnitudes of each wavelet.

**Table 10:** Acoustic signal pre-processing and feature extraction.

Authors	Processing Methods	Feature Extraction
Zhang et al. [61]	Wavelet packet decomposition.	Time domain analysis, Teager energy operator, and cross-correlation.
Rocha et al. [59]	Fast Fourier Transform (FFT).	Means of the magnitude.
Ericeira et al. [50]	Normalized Data acquisition in the time domain and Fast Fourier Transform.	Mean, median and standard deviation.
Liu et al. [8]	Frame the audio using the hamming window, DFT, and take the logarithm of the amplitude spectrum of DFT data.	MFCC.
Yang et al. [40]	First three seconds and the last three seconds are removed from each 20 s audio signal.	The mean value, the peak value, the root mean square (RMS), the variance, the standard deviation, the skewness and the kurtosis and zero crossing rate, MFCC used PCA and autoencoder to extract features automatically.
Peng et al. [24]	Wavelet packet transform.	Energy spectrum, standard deviation, mean, etc.
Xiao-ping Jiang and Guan qiang Cao [41]	Wavelet transform and normalization energy of each layer.	A sum of energy of each frequency band.

**Table 11:** Papers selected for reviewing FD in belt conveyor idlers.

References	Year	Publication Venue	Publication Type	Method	Signal Type
Bortnowski et al. [44]	2022	Eksploatacja I Niezawodnosc-Maintenance and Reliability.	Journal.	LSTM.	**Vibration signal**
Ravikumar et al. [48]	2021	Advances in Smart Grid Technology.	Book Chapter.	Random Forest Algorithm.	
Ravikumar at al. [45]	2019	Measurement.	Journal.	k-star algorithm.	
Ravikumar at al. [47]	2014	International Conference on Computational Intelligence and Advanced Manufacturing.	Conference.	SVM.	
Li et al. [4]	2013	Advances in Mechanical Engineering.	Journal.	Wavelet Packet Decomposition and Support Vector Machine.	
Wijaya et al. [12]	2022	Measurement.	Journal.	Iforest.	
Ravikumar et al. [46]	2018	Emerging trends in engineering, science, and technology (ICETEST).	Book section.	Artificial neural network and Naïve Bayes algorithm.	
Muralidharan et al. [58]	2014	Measurement.	Journal.	Decision tree.	
Wijaya et al. [60]	2021	Structural Control and Health Monitoring.	Journal.	Wavelet transform and artificial neural network.	
Roos and Heyns [23]	2021	Mining and Mineral Engineering.	Journal.	Wavelet package decomposition and artificial intelligence, SVM.	
Ericeira et al. [50]	2020	International Joint Conference on Neural Networks (IJCNN).	Conference.	RF and MLP.	**Acoustic signal**
Liu et al. [8]	2020	Advanced Powder Technology.	Journal.	Decision tree Gradient boosting.	
Yang et al. [40]	2020	Neurocomputing.	Journal.	SVM, *K*NN, ANN, CNN.	
Peng et al. [24]	2020	Control Engineering Practice.	Journal.	CNN.	
Xiao-ping Jiang and Guan qiang Cao [41]	2015	International Conference on Natural Computation (ICNC).	Journal.	Neural network.	
Rocha et al. [59]	2021	Journal of Intelligent and Robotic Systems.	Journal.	RF.	

**Table 12:** Vibration FD methods used in belt conveyor idlers via ML models.

Authors	Detection Methods	Accuracy	Main Findings	Advantages	Disadvantages
Li et al. [4]	WPD and SVM techniques	100%	The results of an experiment performed on a belt conveyor in a coal mine demonstrate that the proposed system can find faulty idlers with limited sensors.	An SVM classifier can correctly identify normal conditions and several faulty conditions.	The computation time of the classification results in a delay in the detection of faults.
Muralidharana et al. [58]	Statistical features and decision tree algorithm	99.52%.	The results of this study may not be generalizable to all cases. Nevertheless, the methodology employed will serve as a guide for future research in this area.	Simple to understand and interpret for feature selection and classification.	The decision tree provides more information about the classification of faults. It can result in trees that are overly complex and are not able to generalize the data well.
Ravikumar et al. [46]	Statistical features and Artificial neural network and naïve Bayes (NB) algorithm	**ANN:** 90% **NB**: 95%	Results from artificial intelligence and NB demonstrate the accuracy of the NB performs better than the ANN algorithm in predicting self-aligning conveyor roller failures and assessing their lifetime.	NB performs better due to its simplicity and assumption of feature independence.	Since the number of faults collected is limited, the proposed method cannot be generalized.
Bortnowski et al. [44]	LSTM Autoencoder model	90%	The use of the autoencoder facilitated the automation of damage detection, which is invaluable when assessing the operation of long-distance conveyor routes.	The proposed method was able to detect the location of potential roller damage based on the change in the average peak frequency over time and spectral autocorrelation.	Low-frequency signals related to the specific operating conditions were regarded as potential sources of damage when they were simply sources of signal interference.
Roos and Heyns [23]	Wavelet package decomposition and artificial intelligence	100%	The use of SVMs for vibration monitoring in-belt systems for conveyor idler bearings is strongly recommended, together with WPDs for preprocessing the signals from the bearings.	SVM can identify bearings that are in the early stages of failure, even with added payloads, compared to ANN.	A certain kernel function may be more accurate in classifying some data sets in SVM. The impact of payloads on signal clarity affected the accuracy of the proposed methods.
Ravikumar et al. [45]	Statistical features and k-star algorithm	91.7%	It is found that statistical features and K-star algorithms are effective tools for detecting faults in self-aligning troughing rollers on bulk material handling belt conveyors.	Since k star is an entropy-based algorithm, it can handle a wide range of complex data sets better than ANN.	A new instance of a class is assigned by comparing the closest existing instance with the new one using a distance metric, which is inefficient in terms of memory usage.
Ravikumar et al. [48]	Statistical features and random forest algorithm	90.2%	Random forest results summarize the accuracy of the algorithm in terms of predicting self-aligning conveyor roller failures as well as assessing the lifetime of the conveyor rollers.	The accuracy of Random Forest is generally high.	Random forests can be computationally intensive depending on the data collected.
Ravikumar et al. [47]	Statistical Features and Support Vector Machine	98.08%	According to SVM results, the algorithm accurately predicted self-aligning conveyor roller failures and assessed conveyor roller life expectancy.	An advantage of the SVM Algorithm is that it can handle multiclassification tasks.	It can be difficult to select the appropriate Kernel functions.
Wijaya et al. [60]	Wavelet transform and artificial neural network	99%	By adopting the proposed fault detection scheme, fault identification and classification can be accomplished accurately and unaffected by changes in operating modes, such as conveyor belt speed.	Three idler conditions tested using ANN provided more than 99% classification accuracy even with varying belt speeds.	Due to the limited number of faults and the inability to simulate the effect of loading on the conveyor frequency signatures, the proposed method was untrustworthy.
Wijaya et al. [12]	A high-frequency energy and envelope spectrum and IForest method are used to determine fault location	90%	According to the study, faulty idlers significantly increased high-frequency energy with a bearing fault detected through envelope analysis.	Most anomalous data points were detected with a 90% reduction in analysis time.	The disadvantage of this method is that data were directly divided into half rather than randomly determining where to split the data.

**Table 13:** Acoustic FD methods applied to belt conveyor idlers via ML Models.

Authors	Detection Methods	Accuracy	Main Findings	Advantages	Disadvantages
Ericeira et al. [50]	The ultrasound sensing combined with RF and MLP machine learning techniques and signal pattern recognition.	Four experiments using different numbers of samples. The best result was achieved in the fourth experiment by extracting from the FFT applied in every 5 s of the 20 recordings divided into 40 parts the MLP10, which in one case attained 89.47% of correctly classified instances.	The results show that the detection performance depends on the features they use to input the classifier.	When frequency domain features are used with more data, the proposed methods demonstrate the best accuracy.	To achieve the highest degree of accuracy, it is necessary to tune the number of trees in RF, the number of neuron layers in MLP, etc.
Liu et al. [8]	MFCCs as features and GBDT algorithm for classification.	Detection accuracy of 94.53%.	The proposed MFCCs and GBDT approach is a viable method for detecting idler roll failures based on sound signals.	A Gradient Boost algorithm with self-learning automatically determines which MFCC feature to apply at which step and what threshold value to determine for this feature.	Window size for extracting MFCC significantly impacts the accuracy performance of GBDT models.
Rocha et al. [59]	For the detection of roller failures, a fast Fourier transform and means of the magnitude of the sound signal are used along with a random forest algorithm.	The trained model has an accuracy of 95% in identifying damaged bearings noise correctly.	Test results demonstrated that ROSI could stand up to harsh operating conditions while carrying out all necessary inspection tasks in a mining site, establishing it as a disruptive solution for belt conveyor.	The RF is simple to use and shows better results.	An unblanched dataset was used to train the method.
Yang et al. [40]	DNN, DCNN, SVM and *K*NN.	The accuracy is 90% *K*NN. The detection accuracy of SVM is 91.9% DNN: The average accuracy is 94.4% DCNN: The classification accuracy is 98%.	Based on the results, the fault detection system works very well for roller fault detection, with an accuracy rate of more than 90.0%.	Models based on deep learning produce better results than traditional models.	Developing a deep learning model requires a large amount of data to train and build a robust model.
Peng et al. [24]	Wavelet packet transformation and CNN have been used.	The classification accuracy rate of the mean as a feature is 86%, and the classification accuracy rate of the standard deviation as a feature is 93%.	According to the experiment results, using the standard deviation as the data feature is more effective than the mean in detecting roller faults.	A CNN can handle a large amount of input data and take into account the location information between the data.	After extracting wavelet packet transformation, data from the lowest frequency band can significantly affect CNN performance.
Xiao-ping Jiang and Guan qiang Cao [41]	Wavelet transform and Neural network	The accuracy rate can reach more than 96%.	In belt conveyors, fault characteristics are contained in fault sounds and can be obtained by adding the energy of each band after the wavelet transform has been applied.	The neural network can easily recognize and classify faults.	The accuracy of the method is affected by environmental noise and the sound of the belt conveyor.

**Table 14:** Acoustic FD methods applied to various industrial systems.

Methods	Advantages	Disadvantages
MSAF	This method can effectively detect faults in electric motors by analyzing acoustic signals.	MSAF methods require the selection of parameters and the number of groups to be determined in advance.
SMOFS	This method helps diagnose faults accurately by using an iterative process to identify relevant frequency components.	As with the MSAF, SMOFS methods require a prior selection of parameters and groupings.
MSAF-17-MULTIEXPANDED-FILTER-14	More accurate results with greater resolution and detail in the signal by using 14 bandwidth and 17 frequency components.	The results of the recognition process depend on the training samples.
SMOFS-22-MULTIEXPANDED	Useful for early fault diagnosis in rotating machines, both electrical and mechanical.	The acoustic signals in this method may overlap and merge, causing problems in analysis, such as reflections and overlapping waves.
MSAF-RATIO-24-MULTIEXPANDED-FILTER-8	High recognition results in diagnosing electrical motors.	Dependence on a lot of training samples and spectral leakage errors in computed frequency bandwidth.
MSAF-RATIO-27-MULTIEXPANDED-4-GROUPS	Implementation of this method is inexpensive and has the potential to be used for a wider range of purposes than just fault detection.	Signals using this method are affected by background noise and reflected sounds.

**Table 15:** Machine learning models in studies.

ML Models	Number of Studies	Studies	Type of Faults	Importance of Faults	Environment of the Tested Result	Efficiency of the Method
Random forests	3	[48,50,59]	Machine faults, including vibration, rotation, noise, sealing, oxidation, and elongation. Four types of conditions: no-fault, ball bearing fault, main shaft fault, and combined faults.	Important.	Laboratory and applied in on mining industry.	Level 5
Support vector machine	4	[4,23,40,47]	Damage to idler bearing and roller, off-center rotation, drum impact.	Important.	Laboratory and applied in on mining industry.	Level 5
Decision tree	1	[58]	Faulty bearings and shafts.	Less important.	Laboratory.	Level 1
Gradient boosting	1	[8]	Artificially defected bearings	Less important.	Laboratory.	Level 1
*K*NN	1	[40]	Broken roller and off-center rotation causing drum collision.	Important.	Laboratory and applied in on mining industry.	Level 1
K star	1	[45]	Faulty bearings and shafts.	Less important.	Laboratory.	Level 1
Isolation forest	1	[12]	Bearing fault, thermo fault, and shell collapse.	Important.	Validated in the real condition in Western Australia for 10 months Laboratory.	Level 3
Naïve Baise	1	[46]	Damage to bearings and shafts.	Less important.	Laboratory.	Level 1
Multilayer perceptron	1	[50]	Abnormal movement of a roller, off-center rotation, excessive noise, inadequate seals, damage from oxidation, and elongation of rollers.	Important.	Validated in real condition.	Level 3
Artificial neural network	4	[23,40,46,60]	Damage to idler bearings, main shaft faults, broken roller, off-center roller rotation, and tire wear.	Important.	Laboratory and validated in real conditions.	Level 5
Convolutional Neural network	2	[24,59]	Stuck and fracture roller.	Important.	Laboratory and validated in real conditions.	Level 5
LSTM autoencoder	1	[44]	Surface of roller tubes, roller unbalance, and radial offset.	Important.	Laboratory and validated in real conditions.	Level 3

Note: Important can lead to device fault; Less-important can lead to incipient fault. Level 5 is the most efficient approach for the efficiency of the method, and level 1 is the least efficient approach.

## Data Availability

Not applicable.
